# An Unusual Case of the “Terrible Triad” in a Transgender Woman

**DOI:** 10.7759/cureus.16869

**Published:** 2021-08-04

**Authors:** Ekta Tirthani, Mina Said, Binita Neupane, Michael Quartuccio

**Affiliations:** 1 Internal Medicine, Rochester Regional Health, Rochester, USA; 2 Endocrinology, Diabetes and Metabolism, Johns Hopkins University, Baltimore, USA; 3 Endocrinology, Diabetes and Metabolism, Rochester Regional Health, Rochester, USA

**Keywords:** euglycemic diabetic ketoacidosis, canagliflozin, hypertriglyceridemia, acute pancreatitis, terrible triad of endocrinology, transgender, estrogen supplements, gallstone pancreatitis, sglt2 inhibitors, transgender female

## Abstract

With the increasing use of sodium-glucose cotransporter-2 inhibitors for type 2 diabetes and heart failure, clinicians need to understand how to treat euglycemic diabetic ketoacidosis (DKA), which is a potential side effect of the medication. The disease triad of euglycemic ketoacidosis, acute pancreatitis, and hypertriglyceridemia (HTG) has complex pathogenesis, and often the trigger of the triad is unknown. Here, we present an unusual case of euglycemic DKA in a transitioning transgender woman on canagliflozin who was treated with 10% dextrose and insulin infusions and apheresis. What makes our case unique is an added layer of complexity with her use of estrogen supplements contributing to HTG and gallstone formation, which could have set off the disease triad.

## Introduction

Although the “terrible triad” of acute pancreatitis (AP), hyperglycemic diabetic ketoacidosis (DKA), and hypertriglyceridemia (HTG) is rare, it has been well documented in the medical literature. With the increasing use of sodium-glucose cotransporter-2 (SGLT2) inhibitors in type 2 diabetes and heart failure, euglycemic DKA is on the rise. Therefore, it is important to know how to treat it. To date, the triad of euglycemic DKA, AP, and HTG has only been reported in two case reports in the medical literature with the use of dapagliflozin. In this case report, we describe an unusual case of euglycemic DKA, AP, and HTG in a transitioning male-to-female transgender woman on canagliflozin and estrogen supplements.

## Case presentation

A 34-year-old transgender woman with a past medical history of type 2 diabetes, hyperlipidemia, and depression presented to the hospital with complaints of abdominal pain, poor appetite, and multiple episodes of nausea and vomiting for two days. The abdominal pain was located in the epigastrium, 8/10 in intensity, radiating to the back, and with no aggravating or relieving factors. She reported that the symptoms had started suddenly after she ate multiple cinnamon rolls and drank five glasses of milk, progressively worsening over two days. She denied any fevers, chills, dysuria, or diarrhea. She denied prior abdominal pain related to fatty foods and did not drink alcohol before the episode. Her medications included oral estradiol 2 mg three times daily, finasteride 5 mg daily, spironolactone 50 mg twice daily, canagliflozin 300 mg daily, metformin 1,000 mg twice daily, atorvastatin 20 mg daily, and lamotrigine 100 mg daily. She endorsed compliance with all her medications until her nausea set in two days prior. She did not have any recent surgeries or gender-affirming surgery. She vaped marijuana occasionally and drank four alcoholic drinks per week.

On arrival at the hospital, she felt dizzy, and her vital signs were: blood pressure 103/50 mmHg, respiratory rate 36 per minute with Kussmaul’s respiration, heart rate 136 beats per minute, temperature 97.7°F, and body mass index 31.3 kg/m^2^. On physical examination, she appeared weak and lethargic with a dry tongue. No abdominal distention or tenderness was noted, and Murphy’s sign was absent. Her blood work showed the abnormalities listed in Table 1.

**Table 1:** Labs obtained on presentation to the hospital.

Lab test	Patient’s labs	Normal range
White blood cell count	27.8 × 10^3^/µL	4–11 × 10^3^/ µL
Glucose	185 mg/dL	65–100 mg/dL
Sodium	128 mEq/L	135–145 mEq/L
Chloride	88 mEq/L	98–108 mEq/L
Serum bicarbonate	<10 mEq/L	22–30 mEq/L
Anion gap	Noncalculable (>25 mEq/L)	4–16 mEq/L
Beta-hydroxybutyrate	7.36 mmol/L	0.02–0.27 mmol/L
Lactic acid	1.4 mmol/L	0.4–2 mmol/L
Lipase	116 U/L	6–51 U/L
Triglycerides	2,142 mg/dl	30–150 mg/dL
Venous blood pH	7.29	7.32–7.42
Hemoglobin A1c	9.6%	<5.6%
Aspartate transaminase	11 U/L	7–37 U/L
Alanine transaminase	15 U/L	10–49 U/L
Alkaline phosphatase	67 U/L	46–116 U/L

**Figure 1:**
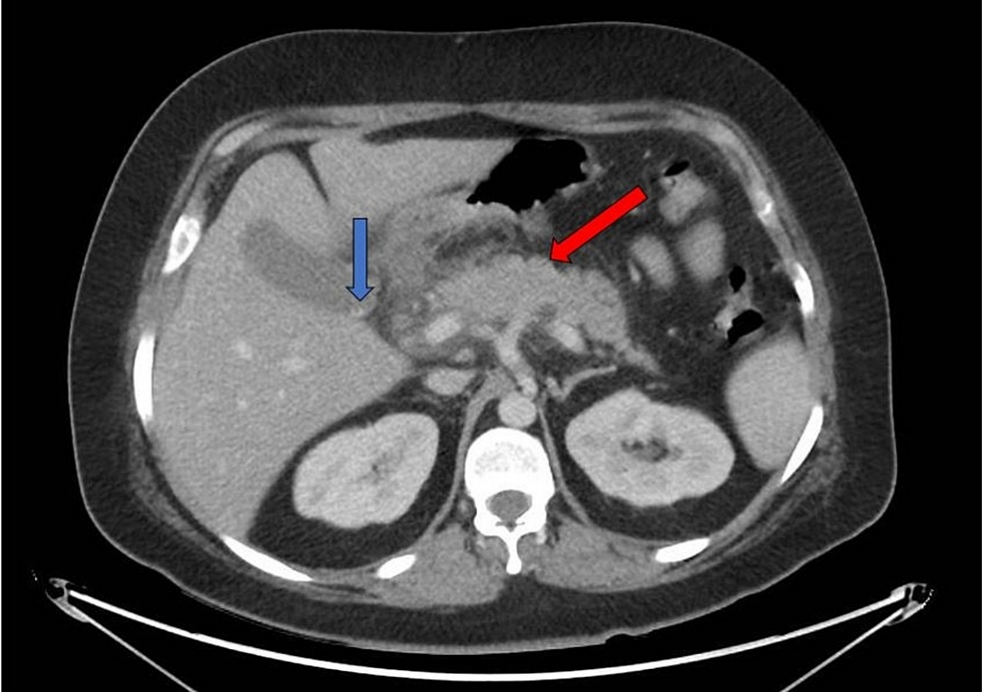
CT scan of the abdomen. The red arrow indicates the edematous pancreatic body and head with moderate surrounding peripancreatic inflammatory stranding and edema suggestive of acute pancreatitis. The blue arrow indicates a small gallstone visualized in the gallbladder. CT: computed tomography

A CT scan of the abdomen showed an edematous pancreatic body and head with moderate surrounding peripancreatic inflammatory stranding and edema suggestive of AP. Gallstones without evidence of choledocholithiasis were also seen on the CT scan (Figure 1).

The patient was diagnosed with an unusual “terrible triad” of AP, HTG, and euglycemic DKA in the setting of canagliflozin and estrogen use and was admitted to the medical intensive care unit. She was treated with boluses of normal saline, and empiric antibiotics with piperacillin/tazobactam were initiated as she met the criteria for sepsis, and her blood cultures were sent for testing. In addition, she was started on a modified DKA protocol with intravenous (IV) insulin along with 5% dextrose in normal saline. Blood sugars fell rapidly, and IV fluids were changed to 10% dextrose. She underwent apheresis to correct the HTG rapidly. Her triglycerides (TGs) fell to 491 mg/dL after the apheresis. It took almost two days for the anion gap to close, metabolic acidosis to resolve, and beta-hydroxybutyrate levels to return to the normal range. She was then transitioned to multiple daily injections of insulin with Lantus and Humalog. Her blood cultures returned negative, following which antibiotics were stopped. It was suspected that the leukocytosis was caused by hemoconcentration and stress.

Once the patient could tolerate an oral diet, fenofibrate 145 mg was started. Canagliflozin was removed from her medication list permanently. In addition, her estradiol was temporarily held until her follow-up appointment with her endocrinologist.

## Discussion

According to the 2021 Gallup survey data, approximately 0.6% of the U.S. population identifies as transgender, which is also supported by an extensive population study by Flores et al. in 2016 [1]. The ratio of transfeminine to transmasculine individuals is 1:1 [2]. Various formulations of supraphysiological doses of estrogen and antiandrogen therapy with finasteride, spironolactone, and cyproterone form the basis of hormonal therapy for transitioning male to female transgender individuals to maintain their secondary sexual characteristics [3,4]. Oral estrogens are notorious for causing AP due to the first-pass effect via portal circulation on hepatocytes. The proposed mechanism involves estrogen decreasing the activity of hepatocyte TG lipase and lipoprotein lipase and increasing the levels of very-low-density lipoprotein. This leads to increased TG-rich chylomicrons, which increase plasma viscosity, causing ischemia and damage to the pancreas [3,5]. Estrogen-induced AP in transgender individuals due to severe HTG has been reported in at least four cases in the medical literature thus far [3,6-8]. Hence, it is recommended that serum TGs should be routinely monitored in transfeminine patients on estrogen therapy, especially in those who have pre-existing HTG [8]. Estrogen also causes cholesterol supersaturation of bile which significantly increases the risk of developing cholesterol gallstones [9]. Freier et al. recently published a case of gallstone pancreatitis in a transgender woman taking estrogen supplements [10]. It is possible that in our patient, a transient obstruction with a gallstone may have contributed to AP, in addition to the more obvious HTG.

SGLT2 inhibitors are being increasingly used for the treatment of type 2 diabetes, heart failure with reduced ejection fraction, and chronic kidney disease. Although once thought to be a rare adverse effect, several case reports have described SGLT2 inhibitor-related euglycemic DKA. In a systemic review and meta-analysis published by Colacci et al. in April 2021, which included >350,000 subjects, it was found that SGLT2 inhibitors were associated with twice the risk of DKA compared to placebo, with the absolute rate being approximately 1 per 1,000 person-years [11]. A multicentric cohort study conducted by Ata et al. showed that canagliflozin (which our patient was taking) carried the highest risk of euglycemic DKA with the highest medical intensive care admission rates [12]. However, there is no consensus regarding the blood glucose level that should be chosen as a cutoff to define euglycemic DKA. While some reports state blood glucose of < 200mg/dL fulfills the criteria for euglycemia, other reports have stated <250 mg/dL should be considered the cut-off, in addition to an increased anion gap (>12 mmol/L), metabolic acidosis (pH <7.3 and bicarbonate <15 mmol/L), and ketonemia or ketonuria [12,13]. In 2017, Burke et al. proposed that the triggers for DKA in patients on SGLT2 inhibitors included surgeries, noncompliance with medication, starvation, pregnancy, ketogenic diets, and dehydration. Euglycemic DKA is especially frequent in patients with long-standing diabetes with more than three months of use of SGLT2 inhibitors [13]. In addition, drug-induced pancreatitis is a rare side effect that has been reported with canagliflozin [14]. Although Gajjar et al. reported HTG due to SGLT2 inhibitor use, it was thought to be an incidental finding rather than true causation due to a lack of pathophysiologic evidence supporting this finding [15].

The “terrible triad” or the “enigmatic triad” of DKA, AP, and HTG is a rare but well-documented entity in the medical literature. Although the trigger is unknown in most cases, once the triad is activated, it has a “domino effect.” While some case reports have shown HTG (with serum TG levels >1,000 mg/dL) to be the trigger of AP which then causes DKA [16], in others, DKA has been suggested as the cause of the severe HTG and AP [17]. In the first case, circulating high serum TG levels are broken down to toxic free fatty acids by pancreatic lipase contributing to pancreatic cell injury with a subsequent insulin deficiency and DKA. In the second case, a primary insulin deficiency causes DKA with an excess of counterregulatory hormones and lipolysis with a decrease in the activity of the enzyme-lipoprotein lipase causing HTG and the resultant AP [8]. However, in a single case report, AP occurred even with mild-to-moderate HTG in a patient with diabetes [18]. Early treatment is recommended because HTG-triggered AP is associated with worse outcomes [19].

**Figure 2:**
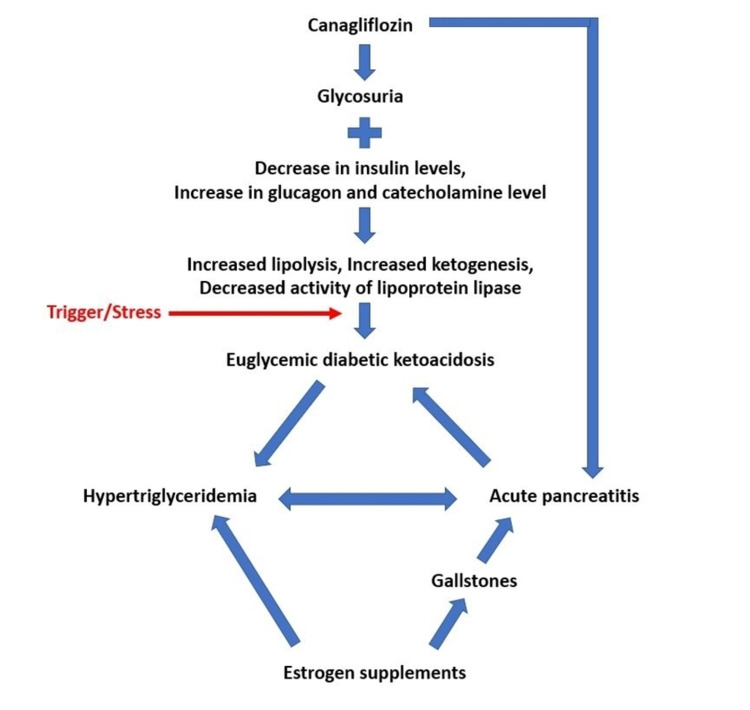
The proposed pathogenesis for the disease triad seen in our patient with the use of canagliflozin and estrogen supplements.

To date, two case reports have been reported in the medical literature describing an unusual “terrible triad” of euglycemic DKA, HTG, and AP with dapagliflozin, but not canagliflozin, though the pathogenesis is thought to be similar due to the class effect [20]. The pathogenesis of euglycemic DKA with canagliflozin use is shown in Figure 2.

There are no specific guidelines for the treatment of euglycemic DKA, especially with concurrent HTG and AP, and it is generally directed by physician experience and reports of treatment success in the medical literature. Generally, the treatment of the disease triad starts by managing the euglycemic DKA with intensive fluid and electrolyte replacement, a continuous IV insulin drip with a concurrent dextrose infusion (up to 20%), and frequent blood glucose checks [8,13,16]. Although therapeutic plasma exchange or apheresis is often used for the management of severe HTG when available, overall, no significant difference has been seen with regular IV insulin therapy with the downside of increasing hospital costs and length of stays [8]. In our patient, we chose apheresis to initially lower the TGs due to the concern that the insulin infusion would take a longer time to correct the HTG as it was being given at a lower rate (0.05 U/kg/hour) than the standard DKA protocol (0.1 U/kg/hour) due to the fear of causing hypoglycemia in this patient with euglycemic DKA. A follow-up with endocrinology was recommended after the disease triad had resolved. Canagliflozin was permanently removed from the medication list and estradiol was held, with a plan for an outpatient cholecystectomy as gallstones may have contributed to the pathogenesis of the disease triad.

## Conclusions

Early diagnosis and treatment of patients with euglycemic DKA, HTG, and AP are essential to stop the domino effect of these disease processes. Patients with the disease triad have significantly higher mortality than patients with just AP. Moreover, the fatality of DKA with the use of SGLT2 inhibitors is higher than that of DKA from other causes. Hence, a good understanding of this disease triad is important, especially in patients on medications such as canagliflozin and estrogen, which can trigger multiple arms of the triad and create a cascade of events like we saw in our patient.
